# Effects of reinforcement during the intertrial interval on temporal discrimination: Location version with rats

**DOI:** 10.3389/fnbeh.2022.956175

**Published:** 2022-09-30

**Authors:** Mario Pérez-Calzada, Oscar Zamora-Arevalo

**Affiliations:** School of Psychology, National Autonomous University of Mexico, Mexico City, Mexico

**Keywords:** temporal bisection, psychophysics, signal detection theory, disruptors, motivation

## Abstract

Different studies on temporal control of behavior have focused on making modifications to experimental tasks by introducing disruptors to know how these manipulations modify temporal control. The aim of this study was to produce changes in temporal discrimination in a temporal bisection task by using a disruptor associated with motivation, which consisted in delivering reinforcement during the intertrial interval (RITI). Four Wistar rats and a pair of duration 2s−8s were used. There were two types of sessions: baseline generalization, where the disruptor was not applied, and RITI generalization, where the disruptive manipulation was applied. The analysis of results consisted of comparing psychophysical parameters, Signal Detection Theory indices, and latencies to start trials of baseline sessions and disruption sessions. The results showed a change in the point of subjective equality, a change in the psychophysical function, an increasing trend in the latencies to start trials on RITI disruption, and no change in the Signal Detection Theory indices. The results highlight the importance of incorporating motivational explanations to theories of temporal control in non-human organisms.

## Introduction

Human and non-human organisms are capable of discriminating temporal stimuli in different duration ranges, from milliseconds to minutes (Reynolds and Catania, 1962; Stubbs, 1968; Catania, 1970; Allman et al., 2014; Cambraia et al., 2020). The temporal bisection task (Church and Deluty, 1977; Penney and Cheng, 2018) is one of the classic procedures to study temporal discrimination.

The temporal bisection task can be divided into two stages, discrimination and generalization. During the discrimination stage, two standard durations are presented, a short and a long stimulus, and then, organisms are trained to categorize these durations as short or long, responding in two response alternatives, for example, in two levers (Church and Deluty, 1977). In the generalization stage, intermediate durations between the two standard durations are also presented to organisms, and the organisms have to classify each of the durations presented as short or long, responding in one of the two response options (Church and Deluty, 1977). The result of this type of temporal learning is a psychophysical function which is commonly plotted as the proportion of long-lever responses as a function of durations. From this psychophysical function, other psychophysical parameters can be obtained, for example, the point of subjective equality (PSE), the differential limen (DL), and the Weber fraction (WF). These psychophysical parameters denote the sensitivity of organisms to the different temporal stimuli to which they were exposed (Stubbs, 1968; Church and Deluty, 1977; Church, 2002; Penney and Cheng, 2018).

In addition, analyses from Signal Detection Theory (SDT) (Green and Swets, 1966; Gescheider, 1997) have also been applied to different procedures within the study of temporal control of behavior (Stubbs, 1968; Raslear, 1985; Wearden, 2008; Akdogan and Balci, 2016b). SDT parameters (e.g., sensitivity and bias) allow for studying the temporal sensitivity of organisms, differentiating perceptual processes that can be associated with detection or decision processes.

To better understand the mechanisms associated with temporal control of behavior, temporal discrimination studies have been carried out incorporating different variables that operate as disruptors. McClure et al. (2010) proposed a classification with two categories for the different disruption manipulations. One category involves those manipulations with pigeons, rats, or humans, in which the stimulus to be estimated is modified, for example, the light or the tone to be estimated may present lesser or greater intensity (Kraemer et al., 1995; McClure et al., 2010; Barrón et al., 2020), or manipulations in which a distractor is presented at the same time that the stimulus whose duration is being estimated (Ward and Odum, 2007). Empirical evidence suggests that when the stimuli have higher intensity, there is a tendency to categorize them as long (temporal overestimation) and the PSE decreases. In contrast, when the stimuli have lower intensity, then they tend to be categorized as short (temporal underestimation) and the PSE increases (Kraemer et al., 1995). On the other hand, manipulations with distractors produce a decrease in temporal sensitivity of organisms which is observed in a flattening of the psychophysical functions (Ward and Odum, 2007; Barrón et al., 2020). However, the results are not entirely conclusive, since psychophysical functions do not always show a clear tendency of temporal underestimation or overestimation (Ward and Odum, 2007; McClure et al., 2010).

The other category (McClure et al., 2010) implies experimental manipulations that modify the motivation of organisms by altering the value of the reinforcers, for example, allowing access to food during the session, extinction of previously reinforced trials (Ward and Odum, 2006; McClure et al., 2009), pre-feeding, and modifications of the magnitude of reinforcers (Galtress and Kirkpatrick, 2009, 2010) with rats and pigeons; or through changes on probabilities, either reinforcement probabilities or probability of presentation of durations to be discriminated with mice and human participants (Akdogan and Balci, 2016a,b; Cambraia et al., 2020). Within this category, mixed results have been obtained; it has been reported that psychophysical functions exhibit an ordered tendency of temporal underestimation or overestimation as a function of experimental manipulations (Akdogan and Balci, 2016a,b; Cambraia et al., 2020). In other studies, however, although a flattening of the psychophysical functions has been found, which suggests a decrease in the discrimination of the intervals to be estimated, the flattening of the psychophysical function has not been completely ordered (Ward and Odum, 2006; McClure et al., 2009; Galtress and Kirkpatrick, 2010).

The experimental findings on temporal control of behavior according to the disruptor classification proposed by McClure et al. (2010) are not entirely conclusive, because the same disruptor can produce either a temporal overestimation, a temporal underestimation, or a flattening of the psychophysical functions. Consequently, to better understand the mechanisms associated with temporal control of behavior and also to know how the behavioral adaptations of organisms to different disruptors are, it is necessary to continue studying the relationship between temporal discrimination and different experimental disruptors.

This research used a temporal bisection procedure with a short-long pair of duration, 2s−8s, with rats as experimental subjects. The experimental manipulation of this study was intended to operate as a disruptor of the motivational processes.

## Method

### Main aim

The main aim was to generate changes in psychophysical parameters on a temporal discrimination task with a disruption of reinforcement during the intertrial interval (RITI).

The experiment involved a temporal bisection procedure with a pair of durations (2s−8s) and with disruption of reinforcement during the intertrial interval (RITI). A temporal bisection task involves two phases, training or discrimination (two durations) and test or generalization (five intermediate durations). This experimental protocol added a disruptive manipulation in the generalization phase.

### Subjects

The animals were four male Wistar rats obtained from the vivarium of the School of Psychology, NAUM. Rats were housed individually and maintained at 80% of their *ad libitum* weight. This experimental protocol followed the guidelines of the official Mexican norm NOM-062-ZOO-1999, Technical Specification for Production, Use and Care of Laboratory Animals.

### Apparatus

Four operant conditioning chambers (MED Associates, Inc., Model ENV-008CT) were used of 29.5 cm (long), 24.8 cm (wide), and 21.0 cm (tall), with two response levers (MED Associates, Inc., Model ENV-110M) for recording responses located on the front wall of the chamber, at 7.0 cm of height, with separation by 1.5 cm of the side walls and separation by 12.0 cm between them. In addition, each chamber also had two triple stimulus displays, each triple stimulus display had three circular lights in red, white, and blue (ultrabrilliant LEDs), each light had 1.4 cm in diameter, and they were separated by 0.5 cm. Each triple stimulus display was located above (5.0 cm) each lever. Only central white lights were used in the experiment.

Each chamber also had a milk receptacle (4.0 cm long, 6.0 cm wide, and 6.0 cm tall) with a white light, in which the reinforcement was delivered. Each milk receptacle was on the front wall in the center, approximately below response levers. A head entry detector was placed into each milk receptacle to record responses. Each chamber also had a liquid dispensing pump (DC12V 5000RPM) connected to the milk receptacle, which delivered 0.1 ml of sweetened milk as a reinforcer. The sweetened milk mixture consisted of 200 ml of water with 15 g of sugar and 30 g of powdered milk, according to Yáñez et al. (2017). The houselight was placed in the middle of the rear wall at 17.0 cm of height and with an intensity of 100 mA. Each chamber had a tone at 4 kHz of frequency and a volume at 70 decibels (dB) to present the durations to be discriminated. The presentation of the stimuli and the recording of responses were carried out through a Dell personal computer with MED-PC IV software, and the personal computer was connected to a MED-PC interface (Med Associates).

### Procedure

#### Neophobia control

There was a neophobia-control session that consisted of presenting the reinforcer to be used (sweetened milk) for 20 min to the rats.

#### Pre-training

All rats associated responding to each of the two levers to obtain a reinforcer that was delivered according to a continuous reinforcement schedule (CRS). All responses on the left and right levers were reinforced, and the light placed in the milk receptacle was turned on during each reinforcement presentation. This schedule ended until rats obtained 40 reinforcers in one session. Once the criterion for the CRS had been fulfilled, rats were shaped with a response-alternation schedule (RAS 1) in which the lit light above one of the levers indicated that reinforcement was available and a single response delivered the reinforcer. The lit light operated as a discriminative stimulus by signaling the location of the reward. The discriminative stimuli were presented randomly in order to distribute the responses of subjects equally between the two levers. The criteria to go to the next scheduled were to obtain 40 reinforcers and that the difference in the number of reinforcers in each lever was not >15%. After reaching the criteria, rats performed RAS 3 and RAS 5 schedules, which presented the same characteristics and restrictions as RAS 1, with the difference that 3 and 5 were the number of responses required to receive reinforcement.

#### Training

Training sessions consisted of 60 trials, ~30 for a short duration (2s) and 30 for a long duration (8s). Left-lever responses after a short duration were categorized as short correct responses, and right-lever responses after a long duration were defined as long correct responses. For half of the subjects, short duration was associated with the left lever and long duration was associated with the right lever as correct responses, and for the remaining subjects, this condition was the opposite. Short and long durations were presented in a semi-random order. All durations were not presented more than four times in a row. Reinforcement for both durations had a probability equal to 1.0 for each correct response (*p* = 1.0) and, once a correct response was issued, an intertrial interval (ITI) with the following durations was presented: 5 s, 10 s, 15 s, 20 s, and 25 s. During ITI, all lights of the operant chamber were turned off. The duration of ITI was presented in random order. An incorrect response or an omission of response (rats had 20 s to issue a response) also led rats to an ITI, and in the following trial, the same type of duration was presented again, that is, correction trials were presented. After a correct response, the occurrence of short or long duration was randomized again. The criterion to finish this training was that the proportion of correct responses for each individual subject was equal to or above 0.80 for three consecutive sessions, or equal to or above 0.75 for five consecutive sessions. The durations to be discriminated were signaled by a 4 kHz tone with an intensity of 70 dB. This tone was used for all the following phases.

During the entire experiment, after the end of ITI values, the stimulus that indicated the time to start a trial was the lit white light in the milk receptacle; therefore, trials were initiated by a head-poking response in the milk receptacle. The time between the start of the lit white light in the milk receptacle and the head-poking response was analyzed as an index of motivation and was called latency to start trials.

#### Training with *p* = 0.75 of reinforcement

Rats had sessions with the same characteristics and temporal learning criteria as in training, but with the only difference that the probability of reinforcement decreased to 0.75 for each correct response.

#### Testing, baseline

Each generalization session consisted of approximately 30 trials for a standard short duration (2s) and 30 trials for a standard long duration (8s) plus 20 trials for intermediate durations, resulting in a total of ~80 trials. Short and long durations were 2s−8s, and intermediate durations were 2.52, 3.17, 4.00, 5.04, and 6.35 s. Intermediate durations had a semilogarithmic difference of 0.1, and each of these durations was presented four times in each session. All durations were presented in a semi-random order; the same duration was not presented more than four times in a row. Reinforcement was presented after a correct answer only for standard durations (2s or 8s); however, correction trials were not used. ITI durations were identical to durations in the training section. Subjects accomplished 10 sessions, which were divided into two blocks of five sessions. After a block was ended, rats received retraining sessions until they achieved the same criterion indicated in the training section.

#### Testing, disruption of reinforcement during the intertrial interval

The characteristics of these generalization sessions were the same as those indicated in baseline sessions, but the manipulation consisted of a disruption of reinforcement during the intertrial interval (RITI) in which one reinforcer was delivered to rats while the ITI was in effect. The reinforcer was delivered in all ITIs during these sessions, always between 2.5 s and 3 s after the ITI was presented.

### Data analysis

The analysis of the results was performed with the data from the 10 sessions of baseline generalization and the 10 sessions of RITI generalization. The following two-parameter sigmoidal function was fitted to the psychophysical function of each subject in order to compare both conditions, refer to Equation 1:


(1) $$p(y)=\frac{1}{1+e^{\left(\frac{-t-x0}{b}\right)}}$$

where *t* is the duration of the stimulus, *1* is the maximum value of the function, *x0* is the duration of stimulus at which the sigmoidal function has raised half of its height, and *b* is the slope parameter.

Once the parameters of the function were calculated, PSE [temporal duration at which *p*(LONG) = 0.5], DL [time at which (LONG) = 0.75 minus time at which *p*(LONG) = 0.25, divided by 2], and WF (DL divided by PSE) were obtained. A lower PSE value could suggest overestimation, and a higher PSE value could suggest underestimation of the durations to be discriminated. In general, lower values of DL and WF indicate better discrimination of the temporal stimuli.

Before fitting the two-parameter model to the data, an asymptote correction was carried out according to Ward and Odum (2007), refer to Equation 2:


(2) $$\begin{array}{l} p\left(R|A\right)=\frac{p\left(R\right)-p\left(R_{L}\right)}{p\left(R_{U}\right)-\;p\left(R_{L}\right)} \end{array}$$

where *p(R|A)* corresponds to the probability of a long response when there is complete stimulus control, *p(R)* equals the probability of a long response in each duration of the psychophysical function, *p(R*_*U*_*)* corresponds to the value of the higher asymptote, and *p(R*_*L*_*)* equals the value of the bottom asymptote. This correction implies that when there is complete stimulus control, then the values for durations of 2s and 8s in the acquired proportion of long responses should be 0 and 1.0 for each duration. If the correction is applied to the data and the described values are not found, this could suggest that the timing behavior does not exhibit a complete stimulus control.

A second analysis was performed according to SDT (Green and Swets, 1966). In this analysis, the first three durations used in the generalization task were categorized as signal (2.0, 2.52, and 3.17 s) and the last three durations were categorized as noise (5.04, 6.35, and 8.0) (Akdogan and Balci, 2016b). Since experimental evidence suggests that the PSE is close to the geometric mean of the reference durations (Church and Deluty, 1977), it was decided to exclude the fourth duration of the stimuli (4s) (Akdogan and Balci, 2016b). Hit and false alarm rates were quantified; then, nonparametric indices of sensitivity (A′) and response bias (B″) were obtained (Stanislaw and Todorov, 1999; Akdogan and Balci, 2016b).

A′ ranges from 0 to 1.0, where a value of 1.0 indicates perfect temporal discrimination at short and long durations while a value close to 0.5 indicates null temporal discrimination of those durations. B″ ranges between −1.0 and 1.0, where a negative value indicates a greater trend of short responses made (liberal criterion), a positive value indicates a greater trend of long responses made, and a value equal to 0 suggests the existence of no response bias. A′ and B″ indices were obtained for each subject.

Finally, since it was established that each trial would begin only until rodents crossed the sensor of the head entry detector placed in the milk receptacle, an analysis of the latencies to start trials was carried out. A shorter or longer latency to start the trials would suggest a high or low level of motivation of the organisms to carry out the task.

Comparisons of the psychophysical parameters, the SDT indices, and the latencies to start trials at baseline and on RITI manipulation were carried out.

## Results

**Figure 1:**
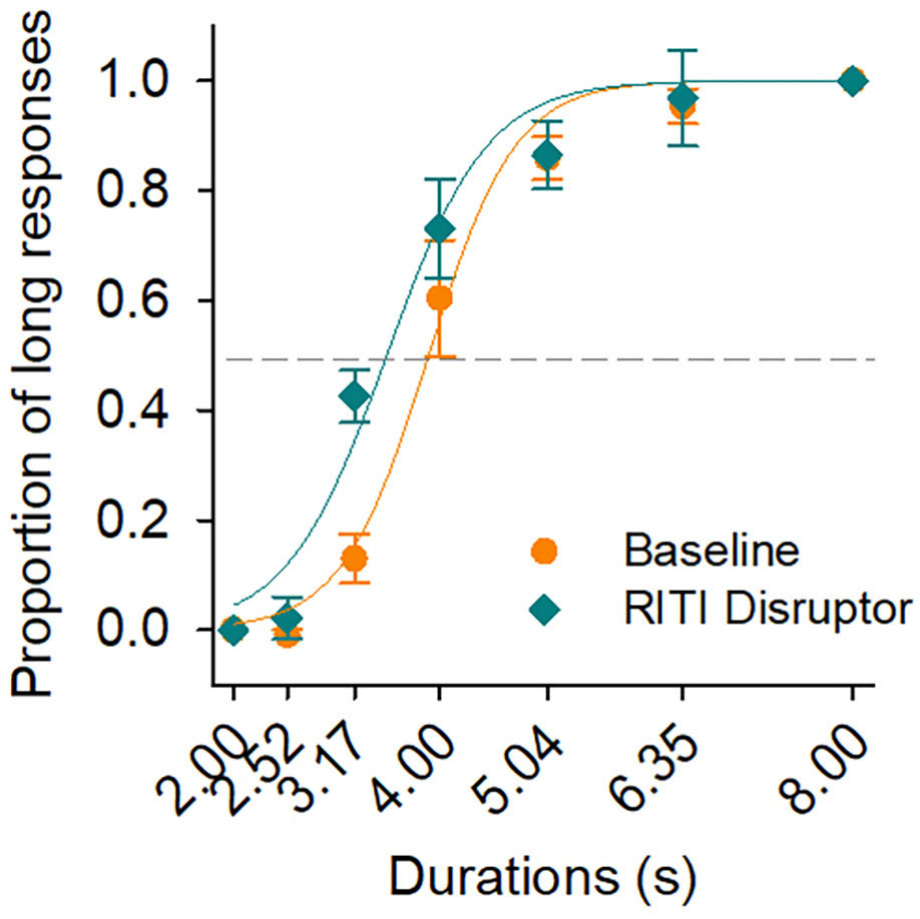
Psychophysical functions at baseline and on disruption of reinforcement during the intertrial interval (RITI). Psychophysical functions at baseline (orange circles) and on RITI manipulation (green diamonds). The continuous line in orange color describes the best fitting of the sigmodal function for baseline, and the continuous line in green color describes the best fitting for RITI manipulation. Vertical bars represent SEM.

**Figure 2:**
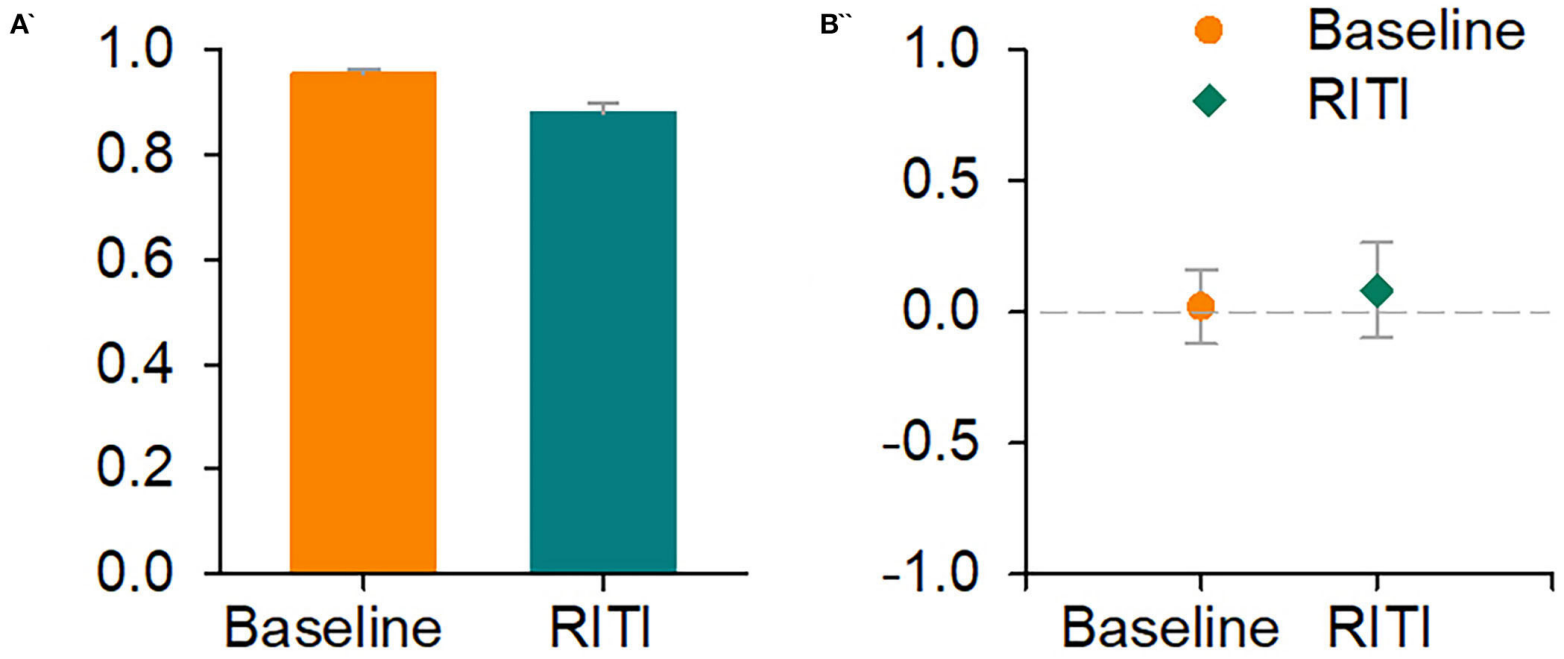
Signal Detection Theory-based analysis: Indices of sensitivity **(A′)** and response bias **(B″)**. Left panel shows **A′** at baseline (orange bar) and on RITI (green bar). The right panel shows **B″** at baseline (orange circle) and on RITI (green diamond). The vertical bars of both panels represent SEM.

Data, parameters, and indices, in Figures 1, 2 and Table 1, correspond to baseline generalization (baseline condition) and RITI generalization (RITI manipulation) of all subjects.

**Table 1:** Point of subjective equality (PSE), difference limen (DL), and Weber fraction (WF) values for the average of all subjects in both conditions, baseline and RITI disruption.

**Parameters**	**Conditions**	
	**Baseline**	**RITI disruption**
PSE	3.92 ± 0.18	3.57 ± 0.24
DL	0.43 ± 0.07	0.56 ± 0.17
WF	0.10 ± 0.01	0.15 ± 0.03

Mean ± SEM.

### Psychophysical functions

Figure 1 shows two psychophysical functions with the average of all subjects. The psychophysical function with orange circles corresponds to the baseline condition and that with green diamonds corresponds to RITI manipulation. The psychophysical functions show the proportion of long responses as a function of the durations to be discriminated. Figure 1 also shows the best fit of the two-parameter sigmoidal function for the data of each condition (continuous line in orange color for baseline and continuous line in green color for RITI manipulation). The R-squared values of the best fitting of the sigmoidal functions were 0.98 and 0.97 for baseline and RITI manipulation, respectively. Vertical bars represent the standard error of the mean (SEM) in both psychophysical functions.

Figure 1 shows that both psychophysical functions increased monotonically as the duration increased. The psychophysical function for RITI showed a shift to the left compared to the psychophysical function at baseline. A repeated measures ANOVA was performed on both psychophysical functions with the variables of Condition (baseline and RITI) and Duration (seven durations). A significant effect of Duration (*F*_(6, 18)_ = 116.20, *p* < 0.00, $\eta_{p}^{2}$ = 0.97) and a significant interaction of Condition and Duration were found (*F*_(6, 18)_ = 10.47, *p* < 0.00, $\eta_{p}^{2}$ = 0.77). A main effect of Condition was also found (*F*_(1, 3)_ = 26.71, *p* = 0.01, $\eta_{p}^{2}$ = 0.89).

### Temporal discrimination parameters

Table 1 shows the averages and SEM of the psychophysical parameters of PSE, DL, and WF of all subjects for both conditions (baseline and RITI manipulation).

In Table 1, the PSE value was lower on RITI manipulation compared to baseline. A repeated measures ANOVA was performed for PSE values in both conditions, and a significant decrease effect was found (*F*_(1, 3)_ = 23.59, *p* < 0.01, $\eta_{p}^{2}$ = 0.88). A repeated-measures ANOVA for DL did not show a significant effect (*F*_(1, 3)_ = 1.31, *p* = 0.33, $\eta_{p}^{2}$ = 0.30). A repeated-measures ANOVA for WF did not reveal a significant effect either (*F*_(1, 3)_ = 2.75, *p* = 0.19, $\eta_{p}^{2}$ = 0.47).

### Sensitivity and response bias

Figure 2 shows the average of the A′ and B″ indices (SDT). In the left panel, the orange bar indicates A′ for baseline, and the green bar indicates A′ for RITI disruption. In the right panel, the orange circle indicates B″ for baseline and the green diamond indicates B″ on RITI disruption. Vertical bars represent SEM for both panels.

In Figure 2, A′ indices of both conditions had values above 0.80; a repeated measures ANOVA did not show significant differences (*F*_(1, 3)_ = 8.22, *p* = 0.06, $\eta_{p}^{2}$ = 0.73). Regarding B″ indices, the values of two conditions were close to zero; a repeated measures ANOVA did not show statistically significant differences either (*F*_(1, 3)_ = 0.29, *p* = 0.62, $\eta_{p}^{2}$ = 0.08).

### Latencies to start trials

This analysis shows the time in seconds that elapsed to start a trial, for all durations in the two experimental conditions (baseline and RITI disruption), on average (with SEM).

The results show that latencies to start trials were higher at RITI disruption compared to baseline, 4.84, and 2.76s, respectively, and there was greater variability (which is reflected in a greater SEM) on RITI disruption compared to the variability at baseline, 1.74, and 0.56s, respectively. A repeated measures ANOVA was performed on both latencies to start trials with the variables of Condition (baseline and RITI), and it was not found a main effect of Condition (*F*_(1, 3)_ = 2.92, *p* = 0.18, $\eta_{p}^{2}$ = 0.49).

## Discussion

The present study used a motivational manipulation (disruption of reinforcement during the intertrial interval, RITI) in a temporal bisection task with the aim of producing changes in Wistar rats' temporal discrimination of auditory stimuli. In this study, during baseline sessions, a reward was given to the rats only after a correct response was issued. In contrast, during RITI disruption sessions, in addition to providing reinforcement after a correct response, the schedule also delivered a reward during the ITI. Therefore, we suggest that by delivering reinforcement during ITI (RITI disruption), the value of the reinforcer delivered after making a correct response decreased. The results of this study did not show significant changes in WF, DL, sensitivity (A′), and response bias (B″), but significant changes in the psychophysical function and the PSE were observed. The results also showed an increasing trend in latencies to start trials on RITI disruption.

The RITI manipulation generated an ordered lateral shift of the psychophysical function, a result that is consistent with the findings from studies with human participants and mice as subjects (Akdogan and Balci, 2016a,b; Cambraia et al., 2020). The psychophysical function of RITI disruption shifted to the left. This finding is not a common result because, in previous studies in which motivational manipulations were applied, such as the extinction of previously reinforced trials, pre-feeding, modifications on reward magnitude, and free access to food during the session, there was a flattening of the psychophysical functions (Ward and Odum, 2006, 2007; Galtress and Kirkpatrick, 2009, 2010; McClure et al., 2009); one explanation of the flattening of the psychophysical function has been the loss of stimulus control (Ward and Odum, 2006; Galtress and Kirkpatrick, 2010; McClure et al., 2010; Galtress et al., 2012). Considering the results found in this study, we suggest that the shift of the psychophysical function on RITI disruption cannot be attributed to a loss of stimulus control because the SDT-based analysis indicated that all durations were correctly detected. Regarding the interaction effect between Condition and Duration, we suggest that the RITI disruption modified temporal discrimination, but specifically for the intermediate durations close to the PSE (the PSE was a parameter in which differences were also found between baseline and RITI disruption).

Signal detection theory analysis (Green and Swets, 1966; Gescheider, 1997) makes it possible to differentiate whether the changes in any detection task can be attributed to the detectability of stimuli or whether the changes occur at the moment of making a decision or a judgment. For example, if the sensitivity does not change, but response bias does change (Stubbs, 1968; Raslear, 1985; Akdogan and Balci, 2016b; Cambraia et al., 2020), then stimuli are being detected correctly, but changes are occurring at the moment of deciding to categorize a duration as short or long. In the present study, the motivational alteration was very strong since each time that an ITI appeared, reinforcement was delivered, and therefore, the reinforcement value was decreased within the experimental session. However, the alteration did not produce changes in the bias response or sensitivity; on the contrary, the SDT analysis suggests that temporal control of the behavior was maintained.

Only in one of the three psychophysical parameters, there was a significant change due to RITI disruption: the PSE. This result is consistent with Raslear (1985) who found a perceptual bias (no changes in response bias nor sensitivity, but a change in PSE) in a temporal bisection task through disruption of stimulus spacing with rats. The change in PSE is also consistent with Akdogan and Balci (2016b), who carried out a differential manipulation of payments at short and long reference durations, with human participants in a temporal bisection task, and also with Akdogan and Balci (2016a), who manipulated the probability of presentation of durations to be discriminated in a temporal bisection task with mice.

The most important result of the present study is the change in PSE that was observed under the RITI manipulation; during baseline generalization, we found that the PSE (3.92) was very close to the geometric mean of the standard durations (4.0), as has been found in several studies that have used the temporal bisection task (Church and Deluty, 1977; Church, 2002; Penney and Cheng, 2018). When the RITI manipulation was in effect, we found a significant decrease in the PSE. Given that the PSE is usually regarded as the criterion to indicate whether a duration will be categorized as short or long, depending on whether it is below or above that criterion (Church and Deluty, 1977), our result suggests that there was a motivationally induced change on temporal discrimination since there was a modification in the criterion to categorize the durations. In the analysis of this possibility, we must acknowledge the small sample size as a potential limitation of our conclusion. On the other hand, it should be emphasized that the decrease in PSE value was reliable across subjects.

The temporal bisection task used in this study (Church and Deluty, 1977) has been categorized as a Location version (McClure et al., 2009); in this type of task, the correct response option (a lever in this case) is always located in the same side of the operant conditioning chamber, left or right. In this study, the results showed a shift to the left in RITI psychophysical function compared to the baseline psychophysical function. This result is consistent with other studies that applied disruptors and used the Location version since those studies found shifts to the left or right but not a flattening of psychophysical functions (Kraemer et al., 1995; McClure et al., 2009, e.g., with Pre-Feeding doubled). In this study, we decided to use the Location version because we wanted to maintain the characteristics of the temporal bisection task according to Church and Deluty (1977).

Another version of the temporal bisection task, in which the correct response option can appear on either the left or right side of the front wall (using the color of keys to issue a correct answer), has been called the Color version (Stubbs, 1968; Ward and Odum, 2006; McClure et al., 2009). The results found using the Color version have shown a flattening of the psychophysical functions (Ward and Odum, 2005, 2006, 2007).

The findings of this study are consistent with those described by McClure et al. (2009) since they suggest that in the location version of the bisection task there is a displacement of the psychophysical functions while in the color version there is a flattening of the psychophysical function.

An explanation for the shift in the psychophysical function found in this study is that there can be a lower susceptibility of modification to the psychophysical functions in a Location version task compared to a Color version task, as McClure et al. (2009) suggested because, in the Location version, organisms can use their own behavior as a guide to issue a correct response (Killeen and Fetterman, 1988; Fetterman et al., 1998; Lejeune et al., 2006).

Another important methodological aspect to consider is whether intermediate durations receive reinforcement or not after a correct response. One of the classic temporal bisection tasks is the task by Stubbs (1968) in which intermediate durations are reinforced after a correct response; however, another classic task is by Church and Deluty (1977) in which the intermediate durations are not reinforced after a correct response, and only the standard durations (short-long) are reinforced. In addition to the above, future studies should take into consideration the duration of ITIs, since small or high values decrease or increase the performance of the subjects in the task; in our study, despite having ITI values that favor high discrimination, the RITI disruption had an effect on psychophysical function and PSE (Roberts and Kraemer, 1982).

Regarding the analysis of latencies to start trials, despite not finding the main condition effect, it is important to highlight the substantial increase in the latencies (2.76–4.84s) and their variability (0.56–1.74s) on RITI disruption compared to baseline. According to Bailey et al. (2016), motivation implies an activational effect on behavior, which can be described through the speed of action; furthermore, according to Bouton (2016), a property that suggests that the behavior is motivated is variability, which indicates that the behavior is guided by the presence of a discriminative stimulus and that the behavior could present less or greater variability depending on the different levels of motivation of the organisms. Therefore, we suggest that the activational effect and the variability property can be represented in the latencies to start trials and SEM, respectively, since during the RITI manipulation the latencies and SEM were higher compared to baseline. That is, the strong trend of increase in latencies and SEM suggests that the RITI disruption modified the motivation of the organisms to perform the procedure.

A temporal bisection task is a procedure that involves processes associated with memory (Penney and Cheng, 2018) since temporal decisions about whether the duration is short or long are made based on the previously reinforced durations of past trials. In this study, through the analysis of DL, WF, and SDT, it can be suggested that the memory process is not being modified since high A′ values were found, and DL and WF values were also found without significant differences, for baseline and RITI disruption conditions, which together indicate adequate detection of short and long durations taking into consideration the previously reinforced durations; furthermore, regarding the moment of making a decision on whether the duration is short or long, the values of B″ in this study indicate that there was no response bias; therefore, the decrease in PSE and the shift of the psychophysical function could be explained through scalar expectancy theory (Gibbon, 1977; Gibbon et al., 1984) due to an increase in pacemaker speed, without changes in the memory or decision process. It is important to note that scalar expectancy theory does not have a parameter directly associated with motivation; in this sense, the results of this study highlight the importance of incorporating motivation as a relevant variable for the theories that explain temporal control of behavior.

The behavioral theory of timing (BeT) is another theory in the study of temporal discrimination. This theory suggests that adjunctive behaviors of organisms allow them to emit responses that exhibit adequate temporal discrimination. BeT proposes the existence of a hypothetical pacemaker that emits pulses, and the pacemaker rate is a function of the reinforcement rate of the experimental session (Killeen and Fetterman, 1988; Lejeune et al., 2006). The pacemaker rate can increase (if the interpulse time decreases) or can decrease (if the interpulse time increases). Evidence indicates that by decreasing the interreinforcer interval during the session, there is also a decrease in the interpulse time (Bizo and White, 1994). The empirical result of an increase or decrease in the pacemaker rate would be a change in the psychophysical function, for example, if a psychophysical function exhibits a higher proportion of long responses, it can be suggested that it is due to an increase in the pacemaker rate. In the present study, the leftward shift of the psychophysical function on RITI disruption compared to the psychophysical function in the baseline is consistent with an increment in the pacemaker rate. Support for this possibility can be found in the study by Morgan et al. (1993) in a temporal discrimination task with 10s and 20s as standard durations since the study by Morgan et al. (1993) found a higher proportion of long responses when a duration of 14s was presented as test duration after the subjects experienced an increase in the rate of reinforcement. Therefore, the results of this study are consistent with previous evidence directly related to BeT (Killeen and Fetterman, 1988; Morgan et al., 1993; Bizo and White, 1994; Lejeune et al., 2006).

The present study demonstrates the advantages of analyzing data from the temporal bisection task using SDT and classical-psychophysics methods. In addition, we also analyzed the latency to start trials as an index of motivation. The complementary nature of these analyses allows us to suggest a conclusion that our manipulation did not affect the stimulus control, but diminished the PSE and generated a change in the psychophysical function due to a motivational disruption.

## Data availability statement

The raw data supporting the conclusions of this article will be made available by the authors, without undue reservation.

## Ethics statement

The animal study was reviewed and approved by Official Mexican norm NOM-062-ZOO-1999, Technical Specification for Production, Use and Care of Laboratory Animals; also followed the APA ethical guidelines, and it was approved by the research Ethics Committee of the School of Psychology of National Autonomous University of Mexico.

## Author contributions

MP-C and OZ-A conceived and planned the experiments, performed the data analysis, and wrote the paper. MP-C carried out the experiments. All authors contributed to the article and approved the submitted version.

## Funding

This research was supported by grants A1-S-11703 from CONACYT and IN303919 from PAPIIT-DGAPA to the OZ-A. The research of this paper was also supported by a Conacyt (CVU 856055) research grant awarded to the MP-C during his Ph.D. studies.

## Conflict of interest

The authors declare that the research was conducted in the absence of any commercial or financial relationships that could be construed as a potential conflict of interest.

## Publisher's note

All claims expressed in this article are solely those of the authors and do not necessarily represent those of their affiliated organizations, or those of the publisher, the editors and the reviewers. Any product that may be evaluated in this article, or claim that may be made by its manufacturer, is not guaranteed or endorsed by the publisher.
